# What to do about resistant bacteria in the food-chain?

**DOI:** 10.2471/BLT.15.030415

**Published:** 2015-04-01

## Abstract

This year’s World Health Day is on food safety. The mass use of antibiotics in animal husbandry is contaminating the food supply with resistant bacteria, causing difficult-to-treat infections while reducing the power of antibiotics to cure human infections. Antoine Andremont talks to Fiona Fleck.

Q: Over 50% of antibiotics consumed globally are given to animals to treat or prevent infections and to boost growth. What are the consequences for human health?

A: Living creatures are heavily colonized by bacteria and many of these bacteria – known as “commensal” – are beneficial for our health. When humans or animals receive antibiotics, most of the medicine is absorbed and goes to the blood, and some of it goes directly to the digestive system, where it kills most of the commensal bacteria – leaving a few bacteria that are resistant and that multiply. Some of an antibiotic that is absorbed by the blood enters the intestinal tract through biliary excretion. So, as a result of antibiotic treatment, human or animal guts contain many more resistant bacteria than those killed by antibiotics. A major consequence for human health is that these resistant bacteria can cause infections – such as urinary tract infections – in the host, which are more difficult to treat than those caused by non-resistant bacteria. When people with weak immune systems, for instance after chemotherapy, intensive care or major surgery, have resistant bacteria in their gut, they may develop severe gut-originating sepsis, which is also difficult to treat. Another consequence for both humans and animals is that large quantities of resistant bacteria are excreted in their faeces, contaminating the environment – and possibly other humans or animals – and may enter the food-chain.

Q: What are the consequences for the food-chain and the safety of our food?

A: When animals that have been given antibiotics are slaughtered, it is impossible to stop all the bacteria – both susceptible and resistant – in their intestine from being disseminated. So meat and other products that enter the food-chain can become contaminated. Although there are country-to-country variations, chickens you currently buy at the supermarket or butcher, are often contaminated with *E. coli* bacteria, which can be highly resistant to antibiotics. When you buy and take home a chicken that is contaminated with resistant bacteria, these bacteria come into contact with your hands as you prepare it, and may spread to kitchen utensils and surfaces. Resistant bacteria in chicken are killed during the cooking process. But if these bacteria contaminate salad or other foods that are eaten raw – they will not be killed. If one member of the family becomes infected with *E. coli-*resistant bacteria, it can easily be passed on to other family members through physical contact. So the consequences for human health are serious.

Q: Are there other examples?

A: There are many examples. One study [in the *Journal of Emerging Infectious Diseases* in August 2013] estimated that more than 1500 annual deaths in the European Union are directly related to antibiotic use in poultry. Another example is the well documented risk of transmission of methicillin-resistant *Staphylococcus aureus* (MRSA) from livestock to farmers, vets and others in close contact to the animals, which can result in severe infections.

Q: When did we first become aware of the problem of antibiotics and resistant bacteria in the food-chain?

A: In the late 1960s the Swann Report in the United Kingdom found that large quantities of resistant bacteria were being excreted by livestock into the environment following antibiotic use in husbandries. At the time no one was worried for one simple reason: new antibiotics were constantly coming on to the market to treat patients. So even if resistance was growing, it was not considered a problem for human health. Since the late 1980s, that has changed because very few new antibiotics have been discovered over the past 30 years. Today we have reached a turning point in the story of antibiotics, where antibiotics are no longer effective in treating infections in increasing numbers of patients. Other warnings by prominent microbiologists, such as Start Levy in the United States were also largely ignored because, when he published his book *The antibiotic paradox* in 1992, even the most resistant bacteria could still be treated with some antibiotics.

Q: When and why did you start studying this field?

A: I became interested in this field as a young physician at the Institut Gustave-Roussy cancer centre, when I started to study resistant intestinal bacteria in the mid-1980s. Together with Cyrille Tancrède, my boss at the time, we found that after chemotherapy, severely immunosuppressed patients were more likely to become infected with their own intestinal bacteria, and that the more antibiotics they took, the more their intestinal bacteria became resistant to antibiotics. We tried to understand how this happened and whether we could stop this process. That led to a series of experimental and clinical investigations that I have pursued since, including in my current work. My laboratory specializes in studying the effect of antibiotics on the dynamic of antibiotic resistance in the intestinal tract. 

Q: What have you discovered? 

A: A key point is that bacteria that populate the intestinal tract of animals and humans are very similar, if not identical, and they can exchange information carried in genes about resistance to antibiotics. So when resistance occurs in animals, it can affect human intestinal bacteria. This is why it is so important to reduce antibiotic use in animals to reduce resistance in humans. That does not mean that we should ignore antibiotics used in humans, if we want to control resistance. But more efforts are needed to slow antibiotic resistance in animals because the quantity of antibiotics used in animals is so much greater, there are many more farm animals than humans, and because the rationale for using antibiotics in animal husbandry is profit-driven rather than health-oriented.

“The rationale for using antibiotics in animal husbandry is profit-driven rather than health-oriented.”

Q: Which food stuffs are contaminated with resistant bacteria?

A: Most types of industrially raised animals receive antibiotics to some extent, although there are major differences between species and practices. Chicken, pork, rabbits and farmed fish receive a lot. In 2014, the French consumer association called *Que Choisir* found that a large proportion of chickens and turkeys in France were contaminated with bacteria that were resistant to third generation cephalosporins – one of the antibiotics that need to be protected as a final line of defence when other antibiotics have failed in humans. Milk is an interesting case. The dairy industry has put very strict controls on milk to ensure that there are no antibiotic residues in the milk as these residues could kill the bacteria needed for fermentation that is crucial for products such as cheese and yogurt. If antibiotics are present in milk it cannot be sold, so farmers use it to feed cattle. But while trying to avoid waste, they create a new problem: the intestinal bacteria of the cattle become resistant, and these bacteria are excreted into the environment and enter the food-chain after their slaughter.

Q: What is the agricultural sector doing about the problem of resistant bacteria in the food-chain? How effective is regulation?

A: The regulatory processes are fairly well observed in Europe. For instance, the European Union has had a ban on antibiotics as growth promoters for the past 15 years. But this is not the case in other parts of the world. Australia has banned the use of fluoroquinolones, which are very important antimicrobials for human medicine, in animal husbandry. As a result, resistance to fluoroquinolones in foodborne pathogens is very low in Australia, and strikingly lower in human bacteria there too.

*Q: In 2012, the French government launched EcoAntibio, an initiative to reduce by one quarter the use of antibiotics in animal husbandry between 2012 and 2017 in France. What progress is being made? Why do you argue in your book, *Antibiotics: the shipwreck*, that this goal is insufficient?*

A: The plan has been implemented for one year. We don’t have the results yet, but it seems that antibiotic use in animals has been reduced and we are fairly optimistic that it will deliver some good results. But the goal could have been much higher. In the Netherlands, where antibiotics use in animals was as high as it was in France, a 60% reduction was achieved between 2008 and 2012.

Q: By severely restricting antibiotic use in animal husbandry to what extent can we slow the development of antibiotic resistance? 

A: As long as we reduce the use of antibiotics, the damage to ecosystems is reversible. This is borne out by the experience of north European countries, where the levels of resistant bacteria in animals saw steep and rapid declines when antibiotic use in animal husbandry was reduced.

Q: What should consumers and the catering sector be doing to address the problems?

A: No specific measures are recommended for consumers and caterers beyond general hygiene. International standards exist for monitoring the presence of pathogens in foodstuffs and declaring the product acceptable for distribution. The question is: should we detect resistant bacteria in food – just as we do pathogenic bacteria, such as salmonella or listeria? So far there are no regulations to address resistant bacteria in a similar way. This is something governments in collaboration with United Nations agencies, such as WHO, the World Organisation for Animal Health and the Food and Agricultural Organization need to discuss.

“We must re-focus the use of antibiotics on their original purpose: to protect human health and save lives.”

Q: What is the way forward?

A: Only half of the antibiotics prescribed in humans are for people who actually have a bacterial infection. Often they are used in patients with viral or parasitic infections against which antibiotics are not effective. In farms and in agriculture extremely large quantities of antibiotics are given to promote growth and prevent infection in animals, and the combined effect of these factors on the environment and our health is huge. We must re-focus the use of antibiotics on their original purpose: to protect human health and save lives, and we must re-assess whether other uses of antibiotics are justified, so that we can preserve the miracle of antibiotics for future generations.

